# TAMs in Brain Metastasis: Molecular Signatures in Mouse and Man

**DOI:** 10.3389/fimmu.2021.716504

**Published:** 2021-09-03

**Authors:** Michael Schulz, Lisa Sevenich

**Affiliations:** ^1^Institute for Tumor Biology and Experimental Therapy, Georg-Speyer-Haus, Frankfurt am Main, Germany; ^2^Biological Sciences, Faculty 15, Goethe University Frankfurt, Frankfurt am Main, Germany; ^3^German Cancer Consortium (DKTK), Partner Site Frankfurt/Mainz, Frankfurt am Main, Germany; ^4^German Cancer Research Center (DKFZ), Heidelberg, Germany; ^5^Frankfurt Cancer Institute (FCI), Goethe University Frankfurt, Frankfurt am Main, Germany

**Keywords:** cerebral metastasis, brain cancer, tumor microenvironment, tumor-associated macrophages, microglia, tumor immunology, targeted therapy

## Abstract

Macrophages not only represent an integral part of innate immunity but also critically contribute to tissue and organ homeostasis. Moreover, disease progression is accompanied by macrophage accumulation in many cancer types and is often associated with poor prognosis and therapy resistance. Given their critical role in modulating tumor immunity in primary and metastatic brain cancers, macrophages are emerging as promising therapeutic targets. Different types of macrophages infiltrate brain cancers, including (i) CNS resident macrophages that comprise microglia (TAM-MG) as well as border-associated macrophages and (ii) monocyte-derived macrophages (TAM-MDM) that are recruited from the periphery. Controversy remained about their disease-associated functions since classical approaches did not reliably distinguish between macrophage subpopulations. Recent conceptual and technological advances, such as large-scale omic approaches, provided new insight into molecular profiles of TAMs based on their cellular origin. In this review, we summarize insight from recent studies highlighting similarities and differences of TAM-MG and TAM-MDM at the molecular level. We will focus on data obtained from RNA sequencing and mass cytometry approaches. Together, this knowledge significantly contributes to our understanding of transcriptional and translational programs that define disease-associated TAM functions. Cross-species meta-analyses will further help to evaluate the translational significance of preclinical findings as part of the effort to identify candidates for macrophage-targeted therapy against brain metastasis.

## Introduction

Mononuclear phagocytes comprise bone marrow-derived progenitors, blood monocytes, and tissue-specific macrophage populations of embryonic origin (1). Fate-mapping studies in mice revealed that macrophage populations of distinct organs (e.g., lung, spleen, liver, brain, skin) are established early during development and are self-maintained during adulthood (2). The cellular identity of tissue resident macrophages is shaped by the local environment of specific organs (3–5). Moreover, the presence of a diverse range of receptors (6) allows macrophages to receive a broad spectrum of signals and thus contribute in autocrine and paracrine interactions. Hence, this functional plasticity places them at the interface of developmental processes, tissue homeostasis, and immunity (1).

**Figure 1:**
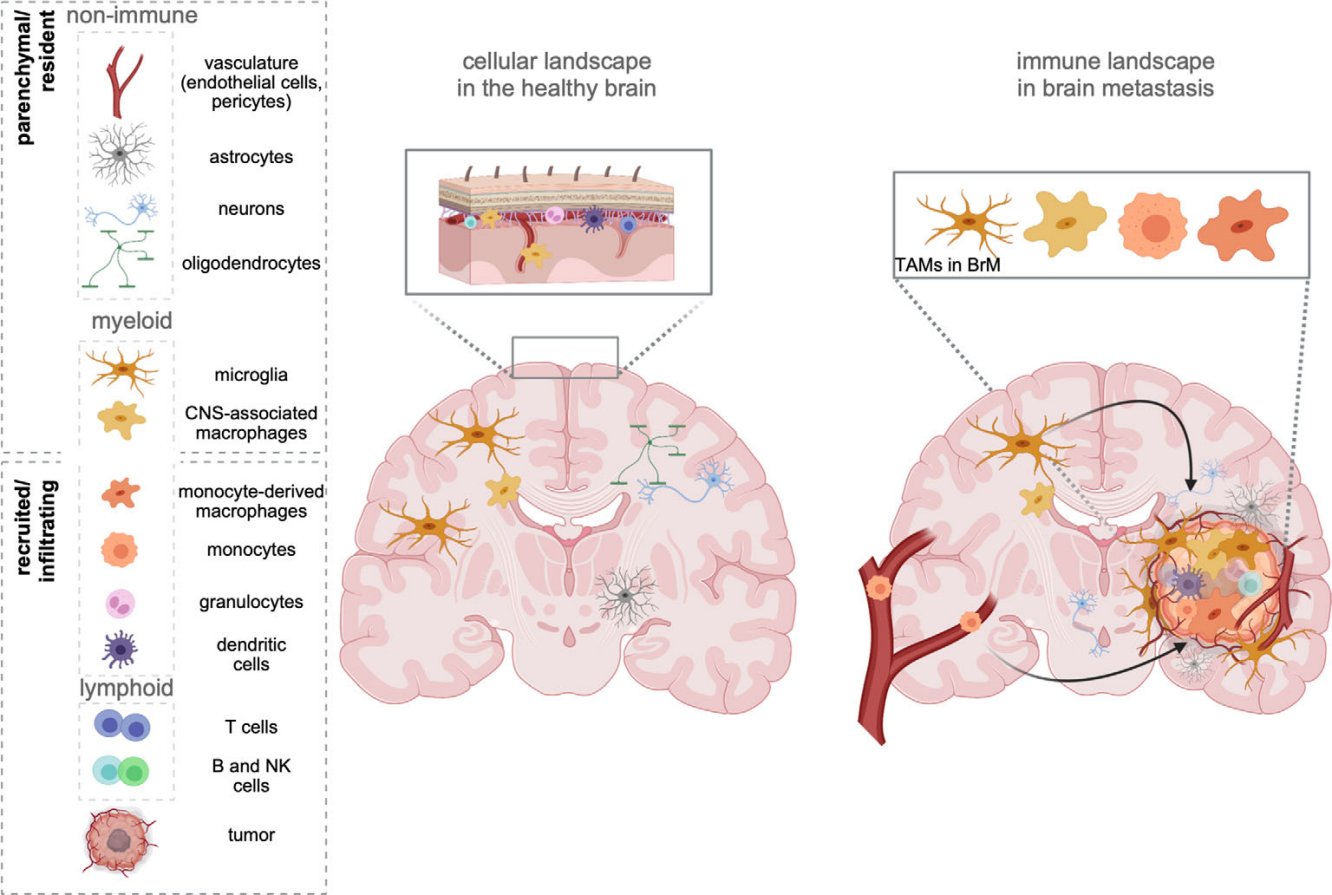
The cellular environment in the healthy brain and BrM. The healthy brain parenchyma consists of resident cell types, including neurons, astrocytes, oligodendrocytes, and cells forming the vasculature (endothelial cells, pericytes). While microglia represent the sole immune cells within the parenchyma, border-associated areas of the brain (e.g., meninges, perivascular areas) harbor every other cell type of the immune system. In contrast, brain metastasis (right) induce the recruitment of all types of myeloid and lymphoid immune cells from the periphery. Tumor-associated macrophages (TAMs) represent a heterogeneous pool of myeloid cells, which consist of brain-resident microglia, as well as monocytes, and monocyte-derived macrophages from the periphery. Recent studies further suggest a partial involvement of recruited CNS/border-associated macrophages (BAMs).

As the sole immune cell type within the immune-privileged brain parenchyma, yolk sac-derived microglia (MG) exert critical functions in immune surveillance and host defense (7, 8). In contrast to the brain parenchyma, where the entry of systemic immune cells is strictly controlled, areas surrounding the brain (e.g., meninges) are constantly patrolled by different classes of lymphoid and myeloid cells (9, 10) (**Figure 1**). In addition to the heterogeneous MG populations that have been identified throughout the brain parenchyma (11, 12), nonparenchymal macrophages found in border regions [= border-associated macrophages (BAMs)] represent a distinct population of central nervous system (CNS) phagocytes (13). Similar to microglia, they derive from yolk sac progenitors during early development (14) and populate the meninges (m), the perivascular areas (pv), and the choroid plexus (cp). Each population is classified based on a specific set of genes, and functional adaptation is driven by local traits. Compared with MG, BAMs exhibit distinct transcriptional signatures (10, 14, 15). Under homeostatic conditions, the structures adjacent to the parenchyma maintain physical and immunological separation of the CNS, but at the same time allow restricted exchange and access of cells and molecules (16).

Neurological disorders disrupt the tissue homeostasis of the brain and lead to the recruitment of cells from the periphery, mostly of myeloid origin (17). Accumulation of myeloid cells within the parenchyma impacts the severity and disease outcome of neurological disorders. Hence, understanding the biology of specific myeloid subpopulations at spatiotemporal resolution is crucial for the development of therapeutic strategies that resolve underlying insults.

A prominent example is the development of brain malignancies. Cerebral or cerebellar tumor formation is accompanied by a massive recruitment of macrophages from the periphery, which together with resident microglia represent the most abundant stromal cell types in primary (18) and secondary (19) brain tumors [brain metastasis (BrM)]. Although every tumor type can metastasize to the brain, the highest incidence is associated to melanoma, lung and breast cancer. Adding up relative numbers, BrM originating from lung or breast cancer contribute to more than 75% of all BrM (20–23).

The brain tumor-associated macrophage (TAM) population consists of cells originating from resident microglia (TAM-MG) and cells of monocytic origin, i.e., monocytes and monocyte-derived macrophages (MDM). However, due to the lack of definitive markers that discriminate both lineages within brain tumors, it was (24, 25) challenging to determine quantitative and qualitative contributions of both TAM populations to brain tumor biology in the past without the need of transplantation models or lineage-tracing approaches. Bowman et al. employed two lineage tracing models in combination with RNA sequencing to identify markers, which reliably allow the discrimination of TAM-MGs and TAM-MDMs in mouse and human primary and metastatic brain tumors. This study led to the identification of the integrin alpha subunit CD49d (encoded by *Itga4*) that is specifically repressed in MG but highly expressed in MDMs. Importantly, this expression pattern remains conserved within brain tumors. In addition, the authors identified CD11a (encoded by *Itgal*) as similarly differently regulated between both major TAM populations (26).

The biggest differences between both TAM subpopulations are determined by their different ontogenetic origin. Since brain TAMs are known to critically influence the progression and outcome of brain tumor biology (24, 25), understanding their quantitative contributions under different conditions and associated putative different functions is key in order to develop novel strategies targeting distinct disease-associated phenotypes in BrM.

## TAMs as Central Part of the Brain Metastasis Microenvironment

TAMs are known to represent a highly abundant cell population in primary and metastatic brain tumors with different quantitative contribution to the myeloid cell pool depending on primary tumor entity (18, 19). However, controversy remained on the functional contribution of macrophage populations depending on their ontogeny. Technical integration of lineage-restricted markers or the use of single cell-based techniques to characterize myeloid cells in brain tumors has significantly broadened our knowledge on TAM heterogeneity in experimental BrM models and patient-derived data from various brain malignancies (**Table 1**).

**Table 1:** Overview of recent studies, examining the tumor microenvironment (TME) of preclinical models of brain metastasis (BrM), and human patient samples.

Reference	Species	Tumor	Main methodology	TAM differentiation		Treatment of individuals	Main targets
				Prior	Post		
Friebel et al. (27)	*H. sapiens*	Various	Single-cell mass cytometry	No	Yes	Treated various (CT, RT, IT)	Protein
Guldner et al. (28)	*M. musculus*	Syngeneic, B2B	CyTOF, CITE-Seq, scRNA seq	No	Yes	Major analyses from untreated	Gene/protein
Klemm et al. (29)	*H. sapiens*	Various	Sorted bulk RNA seq, FCM	Yes, FCM, CD49d		Untreated and treated (CT, RT, IT, others)	Gene
Niesel et al. (30)	*M. musculus*	Syngeneic, B2B	Sorted bulk RNA seq, FCM	Yes, FCM, CD49d		Untreated	Gene
Rubio-Perez et al. (31)	*H. sapiens*	Various	scRNA seq, TCR seq	No	Yes	Treated various (CT, RT, IT)	Gene
Schulz et al. (32)	*M. musculus*	Xenograft, L2B	Sorted bulk RNA seq, scRNA seq, FCM	Yes, FCM, CD49d		Untreated and treated (RT)	Gene

H. sapiens, Homo sapiens; M. musculus, Mus musculus; FCM, flow cytometry; CT, chemotherapy; IT, immunotherapy; RT, radiotherapy; B2B, breast-to-brain; L2B, lung-to-brain.

Two recent studies explored the cellular heterogeneity of myeloid cells in experimental brain metastasis models by multicolor flow cytometry (FCM) integrating CD49d as a marker to distinguish TAM-MG and TAM-MDM. Although approximately 75% of all CD45^+^ cells of the syngeneic breast cancer model 99LN-BrM were of myeloid origin, only 5%–10% of all TAMs were MDMs (30). By comparison, the xenograft lung cancer BrM model H2030-BrM induced stronger TAM-MDM recruitment (32), which constantly increased across different stages of tumor progression leading to 10% and 20% of TAM-MDMs in small or large BrM, respectively (32). Moreover, it was demonstrated that the TAM population within the H2030-BrM model changed in response to whole brain radiotherapy (WBRT), applied as a standard-of-care treatment modality (32). A relative reduction of TAM-MDM contribution to the total TAM pool was observed 3 days after hypofractionated as well as classically fractionated WBRT. Interestingly, this effect was transient and constant reinfiltration resulted in a steadily increasing TAM-MDM population, as examined within the total myeloid cell pool in H2030-BrM at several time points after WBRT. Hence, the application of radiotherapy represents a useful strategy to modulate the TAM pool by causing radiation-mediated cell elimination on the one hand, but enhancing infiltration of naïve cells from the periphery on the other hand. A similar way of interfering with MDM recruitment has been shown in mouse models of glioma in response to radiation (33).

**Figure 2:**
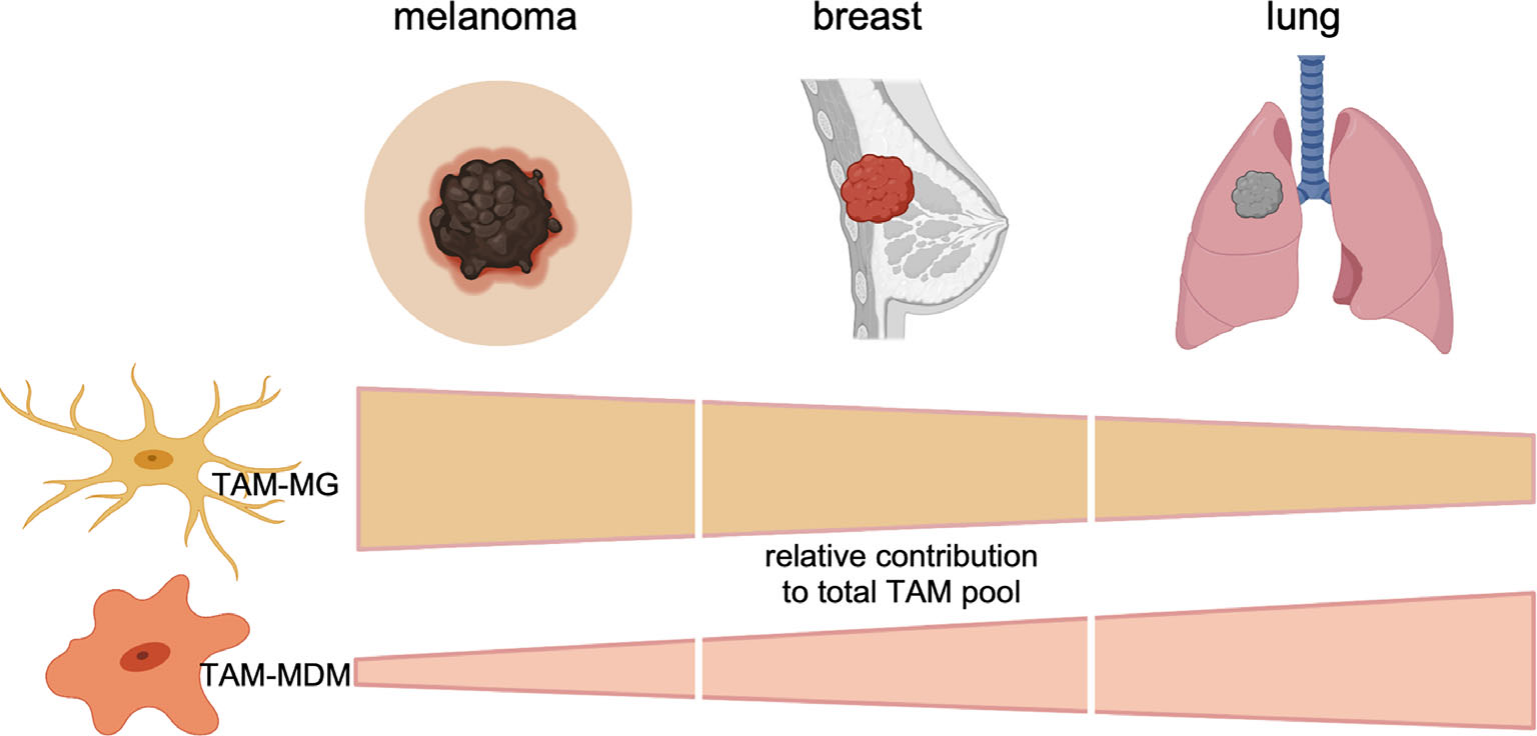
Relative contribution of each TAM subpopulation to the total TAM pool in BrMs derived from melanoma, breast cancer, or lung cancer. Data represent cumulative, relative data derived from preclinical (mouse models) and clinical (human) findings.

Collectively, these data suggest that the TAM pool in preclinical BrM models is highly dynamic. Moreover, recent studies highlighted the contribution of each TAM population to total TAMs. The relative contribution of each TAM population to the total TAM pool is influenced by the primary tumor entity and can be modulated by radiotherapy. TAMs of peripheral origin have been found to be more abundant in recurrent glioma samples upon surgery (34), further illustrating the impact of antitumor therapy on the immune landscape. Interestingly, the diversity of the TAM pool is similarly regulated by the origin of the primary tumor in human BrM (27, 29, 31). Within the studies by Friebel et al. and Klemm et al. the authors performed comprehensive in-depth analysis of patient-derived primary and secondary brain tumor tissue by integrating high-dimensional techniques, such as, FCM, RNA sequencing, or mass cytometry by time of flight (CyToF) to gain insight into cellular and molecular aspects of the brain tumor immune landscape. In contrast to primary brain tumors, BrM induced higher infiltration of myeloid cells from the periphery, and the majority of CD45^+^ cells was composed of neutrophils and MDMs (27, 29). Lower abundance of macrophages from the periphery was observed in melanoma BrM compared with breast and lung cancers (**Figure 2**).

## Molecular Profiles of TAMs in BrM

TAMs are malignancy instructed and have been described as key players at the interface between tumors and cells of the immune landscape. This can be attributed to their high capacity of integrating a broad range of external stimuli, resulting in diverse activation states and highly plastic phenotypes (35, 36). In the following paragraphs, we will provide an overview of molecular alterations observed in both major TAM populations of mouse and human BrM and highlight representative markers that have been identified to be differentially expressed in TAM-MG and TAM-MDM. Moreover, we will discuss candidate factors that have been identified as core signatures of disease-associated macrophages and are commonly up- or downregulated in both major TAM populations. An overview of the representative factors can be found in **Table 2**.

**Table 2:** Selected markers and their regulation within murine (left) and human (right) TAM-MG and TAM-MDMs.

Category	Target (depending on study, referred to gene or protein)	Mouse		Human	
		TAM-MG	TAM-MDM	TAM-MG	TAM-MDM
Microglia lineage	CX3CR1	(28, 30, 32)	(30, 32)	(28, 29)	(27, 29)
	P2RY12	(28, 30, 32)	(30, 32)	(28, 29)	(29)
	SALL1	(30, 32)	Slightly up (30, 32)	(29)	Low (29)
	TMEM119	(28, 30, 32)	(30, 32)	(28, 29)	Unchanged (29)
Macrophage lineage	CCR2	Lower in MG (30, 32)	Higher in MDM (30, 32)	Lower in MG (27, 29)	Higher in MDM but downregulated (27) or upregulated (29)
	CD49d	Lower in MG (30, 32)	Higher in MDM (30, 32)	Lower in MG (27, 29)	Higher in MDM (27, 29)
Antigen presentation	H2-Aa (only mouse)	Lower than MDM, but upregulated (30, 32)	Higher than MG (28, 30, 32)		
	H2-Ab (only mouse)	Lower than MDM, but upregulated (30, 32)	Higher than MG (28, 30, 32)		
	H2-Eb (only mouse)	Lower than MDM, but upregulated (30, 32)	Higher than MG (28, 30, 32)		
	H2-D1 (only mouse)	(30, 32)	Strongly upregulated (30), slightly regulated (32)		
	B2M	(30, 32)	(30, 32) (higher than MG)	Strong upregulation (29)	Slight upregulation and higher than MG (32)
	HLA-A (only human)			(29)	(29)
	HLA-DR (only human)			(27) [high but slightly decreased expression in (29)]	Higher presence than MG (27, 29)
	CD74	(30, 32)	Higher than MG (28, 30, 32)	Slight downregulation (29)	(29)
T cell interaction	CD275/ICOSLG/B7-H2	Unchanged (30) or slightly upregulated (32)	Upregulated (30, 32)	No expression change (29)	(29)
	PD-L1	Unchanged (30) or slightly upregulated (32)	Higher than MG (28, 30, 32)	No expression change but higher than MDM (27, 29)	Upregulated in MDM-3 (27), no expression change (29)
Complement	C1Q	(28, 30, 32) (but higher than MDM)	(28, 30, 32)	Slight downregulation (29)	(29) C1QB high (31)
	C3	(30, 32)	Unchanged (30) or slightly upregulated (32)	(28, 29)	(29)
	C3AR1	(30, 32)	(30, 32)	(29)	Strong upregulation (29)
	C4B	(30, 32)	(30, 32)	Slight upregulation (29)	Slight upregulation (29)
	C5AR1	(30, 32)	(30, 32)	Slight downregulation (29)	Slight downregulation (29)
Cytokine	CCL2	(30, 32)	(30, 32)	(29)	Unchanged (29)
	CCL3	(30, 32)	(30, 32)	(29)	Strong upregulation (29), high expression (31)
	CCL4	(30, 32)	(30, 32)	(29)	Strong upregulation (29), high expression (31)
	IL1A	(30, 32)	(30, 32)	Slight downregulation, high level (29)	Strong upregulation (29)
	IL1B	(30, 32)	(28, 30, 32)	Slight downregulation, high level (29)	Strong upregulation (29), high expression (31)
	TNF	(30, 32)	(30, 32)	Slight downregulation, high level (29)	Strong upregulation (29)
TAM signaling	AXL	(30, 32)	(30, 32)	(29)	(27, 29)
	MERTK	Downregulated (30), unchanged (32)	(30, 32)	Moderate (27)	Partially (27) or strong upregulated (29)
	GAS6	(30, 32)	(30, 32)	(29)	Strong upregulation (29)
Growth factor and ECM organizer	APOE	(30, 32) (higher than MDM)	(28, 30, 32)	Mixed acc. to primary (29), higher expressed than MDM (31)	Unchanged/slightly downregulated (29)
	CTSB	(30, 32)	(30, 32)	Unchanged (29)	Downregulated, but higher levels than MG (29)
	LGALS3	(28, 30, 32)	Not regulated (30) or downregulated (32), higher than MG (28)	Strong upregulation (29)	Slightly downregulated, but higher than MG (29)
	SEMA4B	(30, 32)	Slightly up (30) or down (32)	(29)	(29)
	SPARC	Slightly upregulated (30, 32)	(30, 32)	Strong upregulation (29)	(29)
	VEGFA	Slightly upregulated (30, 32)	(30, 32)	Moderately upregulated (29)	Strong upregulation (29)
Receptors	CD33/SIGLEC3	Downregulated (30), unchanged high expression (32)	(30, 32)	Absent (27)	Down on protein level (27), up on RNA (29)
	CD64/FCGR1	Unchanged (30, 32)	Upregulated (30), no change (32)	(29)	(27, 29)
	CD163	No expression (30, 32)	No expression (30), upregulated (32)	Low (27) or upregulated (29, 31)	(27, 29, 31)
	MARCO	No expression (30, 32)	No expression (30, 32)	Low expression (29)	Strong upregulation (29)
	NR4A2	(30, 32)	(30) (slightly) (32),	(29)	Strong upregulation (29)
	CD206/MRC1	Downregulated (30) or slightly upregulated (32)	(30, 32)	(29)	(27, 29, 31)
	P2RX4	(30, 32)	(30, 32)	Unchanged high expression (29)	Slight downregulation (29)
	TREM2	Slightly upregulated (30, 32)	Strongly upregulated (30, 32)	Slight downregulation (29)	Unchanged high expression (29)
Others	CD209/CLEC4L	No expression (30) or slightly upregulated (32)	High expression (30, 32)	Low (27, 29)	High on MDM-3 (27) or downregulated (29)
	MS4A family members				
	MS4A4C	Slight upregulation (30, 32)	No change (30, 32), upregulated (28)	In *H. sapiens*: MS4A4E, low expression (29)	In *H. sapiens*: MS4A4E, strong upregulation (29)
	MS4A6C	(28, 30, 32)	Unchanged high expression (30, 32); slightly upregulated (28)	In *H. sapiens*: MS4A6E, low expression (29)	In *H. sapiens*: MS4A6E (29)
	MS4A7	(30, 32)	High expression (30, 32)	Slight downregulation (29)	(29)
	S100A family members				
		Higher then MDM in (28): S100a4, S100a6, S100a10	S100a6, S100a10 (32)	(29): S100A4, S100A6 [high in (31)], S100A10 [high in (31)]	(29): high expression level but down in TAM-MDM (S100A4, S100A6)
		Not expressed: S100a4 (30, 32), S100a6 (32); S100a10 [slightly down (32)]		(31): low expression of S100A8, S100A9	(31): slight expression of S100A8, S100A9
		S100a6, S100a10 (30)			
	S100A16	Slightly upregulated (30), downregulated (32)	Upregulated (30), no expression (32)	(29): strong upregulation	(29): slight upregulation
	SPP1	(30, 32)	(30, 32)	High expression, but unchanged in TAM-MG (29)	(29): slight upregulation

Red cell, upregulated; blue cell, downregulated; grey cell, comment within the corresponding cell. In addition, each cell contains information of the respective reference. Note that if not stated otherwise, up/downregulation is referring to the comparison of BrM-associated cells vs. their normal counterpart.

### Transition From Normal to Disease-Associated Cell States

It is increasingly appreciated that tumor-associated immune cells are significantly different compared with their normal cellular counterparts. However, it remains less well understood how normal cells transition into disease-associated cell types upon initial contact to tumor cells and within different stages of tumor progression. Interestingly, Schulz et al. observed that the instruction and education of TAMs represents an early event during formation of the BrM-TME and occurs rapidly after recruitment of resident TAM-MG or peripheral-derived TAM-MDMs. Analyses of transcriptional program in TAMs isolated from small- *vs.* large-stage BrM revealed an almost complete absence of differences in gene expression in each population, suggesting stable transcriptomes during BrM progression (32). However, further dissection of potential transition stages based on single-cell approaches are needed to characterize the acquisition of diseases-associated signatures across a broader range of different stages of tumor progression and to identify the progenitor cells that contribute to tumor-associated myeloid cell pool for more precise comparison. In the following paragraph, we will therefore highlight recent insight on signatures of transition states based on trajectory analyses. Interesting observations on the cellular differentiation route of TAM-MDMs were made in a recent study in which the authors dissected the myeloid cell pool in different stages of murine and human glioma by single-cell RNA-Seq (scRNA-Seq) approaches in combination with lineage-tracing experiments in mouse models (34). By adoptively transferring classical or nonclassical monocytes from Cx3cr1^GFP/+^ mice into *Ccr2*-KO mice harboring orthotopically transplanted gliomas, the authors demonstrated that only classical monocytes were able to differentiate into MDMs within the tumor. If this applies to TAM-MDM in BrM requires further investigation.

### Disease-Associated Transcriptional Signatures of TAM-MG in BrM

Microglia under homeostatic conditions represent a heterogeneous cell population depending on their localization within the brain parenchyma. Moreover, MG heterogeneity is modulated by developmental stages with lower heterogeneity found in adults compared with embryonic stages. Given the inherent MG heterogeneity, it is not surprising that brain pathologies induce an even higher degree of heterogeneity (12, 37), which was demonstrated with single cell RNA-seq for TAM-MG (28, 32). For example, by using t-distributed stochastic neighbor embedding (tSNE), a dimensionality reduction method, of RNA-Seq data from single cells, Schulz et al. reported that the majority of TAM-MG from the H2030-BrM model were contributing to three transcriptionally distinct cell cluster in treatment-naïve BrM. The cluster comprising most of the TAM-MG (cluster 9) was represented by high expression of cytokines (*Ccl3*, *Ccl4*, *Cxcl13*), cathepsin Z (*Ctsz*), the epidermal growth factor receptor 1 (*Egfr1*), as well as MG typical marker (*C1qa*, *Hexb*) (38, 39). The complement member and MG lineage marker *C1qa* was found to be upregulated in TAM-MG in several studies (**Table 2**), whereas other members of the MG-specific “sensome” core signature (*Cx3cr1*, *P2ry12*, *Tmem119*) (11, 38, 39) were consequently downregulated in murine and human TAM-MG (**Table 2**). While an increased expression of *C1q* members in MG belongs to their activation profile (40, 41), downregulation of homeostatic markers most likely is a consequence concomitantly occurring with downregulation of the homeostatic regulatory gene *Sall1*/*SALL1* in murine and human TAM-MG. This further mirrors a classical activation response of MG associated to any damage of the brain, as observed under neuroinflammatory conditions (42).

Once activated and educated by tumor cells, TAM-MG upregulate several markers known to be crucial for inflammation in the injured brain, thereby probably contributing to BrM progression, e.g., *via* exerting immune-suppressive functions. Presumably, this is accompanied with profound metabolic changes as seen in an apparent deregulation of members of solute carrier (*Slc*) genes (32).

Some of the frequently observed markers in MG associated to neurological damage are apolipoprotein E (*Apoe/APOE*) and the triggered receptor expressed on myeloid cells 2, *Trem2/TREM2*, which were highly expressed/upregulated in TAM-MG of murine BrM models, whereas the expression of both members only slightly varied in human TAM-MG. The APOE-TREM2 axis has been described to drive activated MG states in neurodegenerative diseases along with downregulation of homeostatic markers (39, 43). However, especially TAM-MDMs showed elevated expression levels of *Trem2/TREM2* in preclinical BrM models or patient material of BrM derived from various primary tumors (**Table 2**). The contributions of TREM2 and APOE to neurological diseases [e.g., Alzheimer’s disease (AD) or multiple sclerosis (MS)] have been extensively described with regard to MG (44, 45). Importantly, it was shown that targeting TREM2^+^ MG represents an interesting approach to attenuate disease progression. In addition to the broadly studied APOE-TREM2 axis, another key player of MG-mediated neurological dysfunction, and ligand for TREM2, is Galectin-3, encoded by *Lgals3/LGALS3*. Galectin-3 shows a multitude of functions in MG. Elevated extracellular levels within the BrM-TME might drive inflammatory responses in a Toll-like receptor (TLR) 4 binding-mediated self-sustaining manner (46, 47). In line with this, TLR4 expression was found to be upregulated in TAM-MG of H2030-BrM (32). In a recent study, Siew et al. analyzed the contribution of MG-derived Galectin-3 signaling in a mouse model of Huntington’s disease (48). Elevated cytokine levels were attributed to high Galectin-3 signaling, and strategies targeting its expression have been shown to be sufficient in decreasing levels of inflammatory cytokines, thereby ameliorating MG-mediated pathogenesis (48).

While profiling across different conditions revealed that *Lgals3/LGALS3* expression levels are strongly increased in TAM-MG, varying but high levels within TAM-MDMs have been found in murine or human BrM as well (**Table 2**). Similarly, high expression levels were rather associated to TAM-MDMs of murine (49), or human recurrent glioma (34), as revealed in high-dimensional single-cell profiling studies. Therefore, contribution of elevated *Lgals3/LGALS3* levels need to be carefully evaluated in a context-specific manner. Another group of genes (S100 family members) shows an interesting alternating pattern across TAMs in mouse and human BrM. S100 members are small, Ca^2+^-binding proteins, which regulate several cellular functions in an autocrine, or paracrine fashion, and can act as damage-associated molecular pattern (DAMP) (50). For example, S100β has been shown to serve as a noninvasive, astrocytic marker of blood-brain barrier (BBB) integrity and function, also in brain tumors (51), whereas several S100A members have been implicated in neurodegenerative diseases like AD (52). In addition, distinct S100 proteins have been associated to the regulation of inflammatory responses and TAM migration and invasion in tumors (50). Hence, upregulation or high expression especially in TAM-MG of BrM (e.g., *S100A6*, *S100A10*) (**Table 2**) (28, 31), might reflect a central mediator of BrM-associated inflammation. In contrast, in glioma elevated expression level of *S100A6* has been implicated in a transitory TAM-MDM state in mouse and humans (34) or was part of a strong “macrophage signature” (49). Since S100A6, which was implicated in tumor progression in several other cancers (53), can either act on MG in a cell-restricted manner or can be secreted, elevated expression levels of *S100A6* and other S100 members need further examination. To date, it remains unclear if the regulation of S100 members represents a cause or consequence of progressing BrM.

Direct comparison of both TAM populations on the single cell (27, 28, 31), and bulk population level (29, 30, 32) revealed further cell type-restricted molecular profiles in mouse models and human patient samples. TAM-MG showed higher upregulation of distinct proinflammatory cytokines (e.g., *CXCL5*, *CXCL8*, *IL6*) (29), or genes belonging to cell migration, e.g., *Vim/VIM* (28, 31) within a changing environment.

Cathepsins (*Cts/CTS*) encompass a family of proteases known to play several protumorigeneic roles in the tumor context by, e.g., remodeling the extracellular matrix (ECM) (54). In the brain, cathepsin S has been described as BrM-promoting *via* enhancing transmigration through the BBB (19). High expression levels of different cathepsins within TAMs of established BrM further suggest profound remodeling of the TME in outgrowing tumors. Among them, *Ctsd* (28) and *CTSL* (31) exhibited higher expression levels in TAM-MG, while *Ctsb* was strongly upregulated in both TAM populations of different murine BrM models (30, 32), but in human BrM was, together with *CTSW*, rather enriched in TAM-MDM (29). Hence, different highly expressed members of this family of ECM modulators further suggest an involvement for generating a BrM-promoting environment, but specific contributions for/with each TAM population in BrM have not been elucidated yet.

### Disease-Associated Transcriptional Signatures of TAM-MDM in BrM

Recruitment of monocytes to the CNS has been predominantly attributed to the chemokine axis including the receptors Ccr2/CCR2 and Cx3cr1/CX3CR1 (17), which was shown in the brain tumor context using lineage-tracing approaches in glioma mouse models (26, 55). Interestingly, while *Ccr2/CCR2* is dramatically downregulated upon entry into the parenchyma and during the monocyte-to-macrophage transition in glioma (55), and also BrM (27), *Cx3cr1/CX3CR1* levels are upregulated in TAM-MDMs (**Table 2**), whereas the protein was downregulated on the majority of MDM subsets but remained abundant on only a small subset (27). These data suggest that high levels of Cx3cr1/CX3CR1 are partially required to integrate into the CNS parenchyma, since the axis is usually involved in glia-neuronal crosstalk (56). Comparing TAM data for the expression of another potent chemokine receptor known to be involved in cell migration, it became apparent that especially TAM-MDMs possess high levels or strongly upregulated the C3A receptor, *C3ar1/C3AR1* in murine and human BrM (**Table 2**). This suggests the contribution of a conserved mechanism of recruitment *via* the anaphylatoxin/chemokine axis.

Given the fact that bulk analysis usually masks different expression/antigen density levels, Friebel et al. comprehensively dissected the heterogeneity of TAMs derived from different human brain malignancies, and showed that TAM instruction is not a random process, but rather driven by the underlying tumor, both in primary and secondary brain malignancies (27). By combining in total 38 markers for their myeloid panel, the authors captured a broad range of cellular states as depicted by distinct lineage-specific, but also activation markers. Upon merging all TAM CyTOF data from both, glioma and BrM samples, the authors created a detailed relationship and trajectory analyses focusing on abundance of typical monocyte/macrophage markers *in silico*. One of the common features they found was downregulation of CCR2 and the SIGLEC family member, SIGLEC3 (CD33), which represents a transmembrane receptor implicated in pattern recognition and regulation of phagocytosis, and in that regard has been described to be a risk factor for AD (57). Moreover, transitioning cells were found to commonly upregulate the innate immune sensor receptor CD163, together with the TAM receptor MERTK (**Table 2**). Monocytes transition to macrophages through a more common MDM state (termed MDM 1), and finally develop into three distinct MDM subpopulations (MDM 2, 3, 4), and this transition was driven by differential regulation of certain markers, including CD169, CD206 (mannose receptor c-type I, MRC1), CD209 (C-type lectin receptor, CLEC4L), CD38, and PD-L1. Some of these markers were further used in combination with IBA1 to specifically stain for MDMs in the TAM compartment (27). Since not only CD206 but also CD209 usually are associated to alternative macrophage activation, it is not surprising that those markers have been found to be predominantly upregulated in TAM-MDMs of murine and human BrM across different conditions (**Table 2**). MERTK upregulation was found to be gradual, and highest antigen density was allocated to the MDM 4 population (27), whereas bulk RNA-Seq of murine and human TAM-MDMs revealed in general elevated expression levels (**Table 2**). MERTK and AXL, which were also found to be significantly upregulated especially in TAM-MDMs (**Table 2**), represent two of the three TAM (*T*YRO3, *A*XL, *M*ERTK) receptor tyrosine kinases, which are involved in phagocytosis and regulation of inflammatory responses (58). Interestingly, one of the ligands for AXL, *Gas6/GAS6*, was also highly expressed within both TAM populations in murine BrM, but rather upregulated in TAM-MDMs of human BrM samples (**Table 2**). Although GAS6-AXL signaling is present in the healthy CNS, and is associated to phagocytosing MG (59), deregulation can cause enhanced inflammation in the CNS (60). Moreover, this signaling axis can lead to a protumorigenic TME (61) and has been found to be coexpressed in TAMs with high *C1QC* levels in various primary tumors (62).

Despite several differences between the same TAM population within both mouse and human, further commonly regulated markers of TAM-MDMs included *Nr4a2/NR4A2*. This nuclear receptor family is known to control macrophage gene expression during inflammation (63) but is implicated in maintaining normal functions of dopaminergic neurons in the healthy brain. Interestingly, *NR4A2* has been found particularly upregulated in a transitory monocyte population in glioma (34), further suggesting regulatory involvement in inducing inflammatory phenotypes during MDM development.

While certain patterns associated to these inflammatory states of TAMs seem to be conserved between species, other families of proteins are rather restricted to either a species, or a cell type. One interesting group encompasses the membrane-spanning (MS) protein family of MS4A members (**Table 2**). While for some of the family members (e.g., MS4A1 = CD20) their functions are well described, most of them remain poorly understood. In a recent study, Liu et al. generated new lineage-tracing mouse models targeting Ms4a3 (*Ms4a3*^Cre^ and *Ms4a3*^CreERT2^) and validated lineage specificity of this marker, which specifically distinguishes monocytes and granulocytes from embryonically derived resident macrophage populations, including MG, under steady state but also inflammatory conditions (64). MG possess strong expression levels of certain Ms4a members (*Ms4a6b*, *Ms4a6c*, *Ms4a6d*, *Ms4a7*) during early development, while these high expression levels are not found in adult MG or in response to injury. Nevertheless, MG of that specific subpopulation of MG identified during early development cluster also showed overlapping features to BAMs (37). Several genes of the MS4A family appeared in all of the BrM-omics studies among the top regulated genes, and some of them have been described as risk factors for AD (65), including *Ms4a4c (mouse)/MS4A4E (human)*, *Ms4a6c/MS4A6E*, and *Ms4a7/MS4A7*. Interestingly, all of them were found at high expression levels or strongly upregulated across both TAM populations of murine BrM with slightly higher levels in TAM-MDM (**Table 2**). Contrary, in human TAMs, MS4A members were found predominantly upregulated or higher expressed *per se* in TAM-MDMs. Similarly, high and stable expression of *Ms4a7* was reported as part of a core signature for BAMs in steady state and under neuroinflammatory conditions (42). Taken together, this data is in line with the situation in glioma TAMs (34) and further corroborate strong similarities in transcriptional profiles between each TAM population derived from distinct diseases. These findings are strengthened by higher intersect levels of each TAM pool between glioma and BrM (29).

Aiming to describe heterogeneity within certain TAM subsets, all of the recent studies looking into the TME by single-cell approaches confirmed that especially the TAM-MDM pool consists of a more diverse range of cellular states (27, 28, 32). Whereas, Guldner et al. even described BAMs to contribute to the TAM-MDM pool within their model. With respect to identity of cell types within cell populations that were FACS purified prior to transcriptomic analyses, it is important to carefully consider procedures of sample preparation and marker selection. Macrodissection of tumor lesions is critical to reduce the risk of diluting the disease-associated cell pool with normal cell types. Moreover, the use of different marker combinations can lead to different assignment of cell types to certain subpopulations. For example, several commonly used markers to discriminate MG and MDM including CD45, SALL1, and TMEM119 (30, 66) are known to show assimilation of expression levels in both populations in brain tumors. Hence, the choice of marker combinations can lead to differences in population assignment. This determines the classification of subpopulations and consequently significantly affects the respective transcriptional programs. Nevertheless, typical non-MG clusters (TAM-MDMs/BAMs) were shown to be dominated by the expression of genes belonging to antigen processing and presentation particularly associated to MHC class II presentation, including *H2-Aa*, *H2-Ab1*, *H2-Eb*, and *CD74* (**Table 2**) in several independent studies (28, 30, 32). Expression of the MHC class II member HLA-DR in human TAMs was similarly higher in TAM-MDMs (**Table 2**), and upregulated expression can be attributed to the transition from monocytes to MDMs (27), similarly as in the glioma situation (34). Interestingly, this raises the question to which extent each TAM population contributes to T-cell interaction, hence influencing a cancer-promoting, or immunosuppressive TME. Using multiplexed immunostaining, spatial organization of TAMs with respect to T cells was examined in murine (30) and human BrM (29). Both studies found a close proximity of both TAM populations to CD4^+^ and CD8^+^ T cells, yet Niesel et al. observed closer proximity of TAM-MDM to T cells compared with TAM-MG based on discrimination by TMEM119 (30). Moreover, it was observed that PD-L1 was almost absent in tumor-free brains, whereas BrM induced the recruitment of PD-L1^+^ myeloid cells, and levels of PD-L1 were highest among TAM-MDMs (30). On the gene expression level, several costimulatory and also inhibitory markers were found to be present among both TAM populations, whereas most of them were expressed at higher levels in TAM-MDMs (30). Representative comparison of different T-cell regulatory markers across TAMs revealed that both, activating *Icosl/ICOSL*, and inhibitory *Pd-l1/PD-L1* markers are present in TAMs with slight higher levels in the TAM-MDM compartment (**Table 2**). Given the spatial organization of TAMs within the BrM-TME, one can assume that TAM-MGs in the BrM periphery are in contact with T cells at the tumor-stroma interface, while immunosuppression within the BrM core is fostered by TAM-MDMs once T cells have entered the tumor mass. Furthermore, T-cell profiles confirm exhaustion states within the TME (27, 30). Additional immunoregulatory mediators within BrMs predominantly derived from TAM-MDMs were the chemokines *CCL8*, *CCL13*, *CCL17*, and *CCL18* (29). Together, all of them are attributed to an alternative, rather tumor-promoting phenotype (= M2) of macrophages, which is found in many tumors (35). Despite this fact, both TAM-MG and TAM-MDMs upregulate a broad range of inflammatory mediators (29, 32), and hence cannot be classified into the conservative M1–M2 scheme, but rather exist within a continuum, depending on their current local environment.

**Figure 3:**
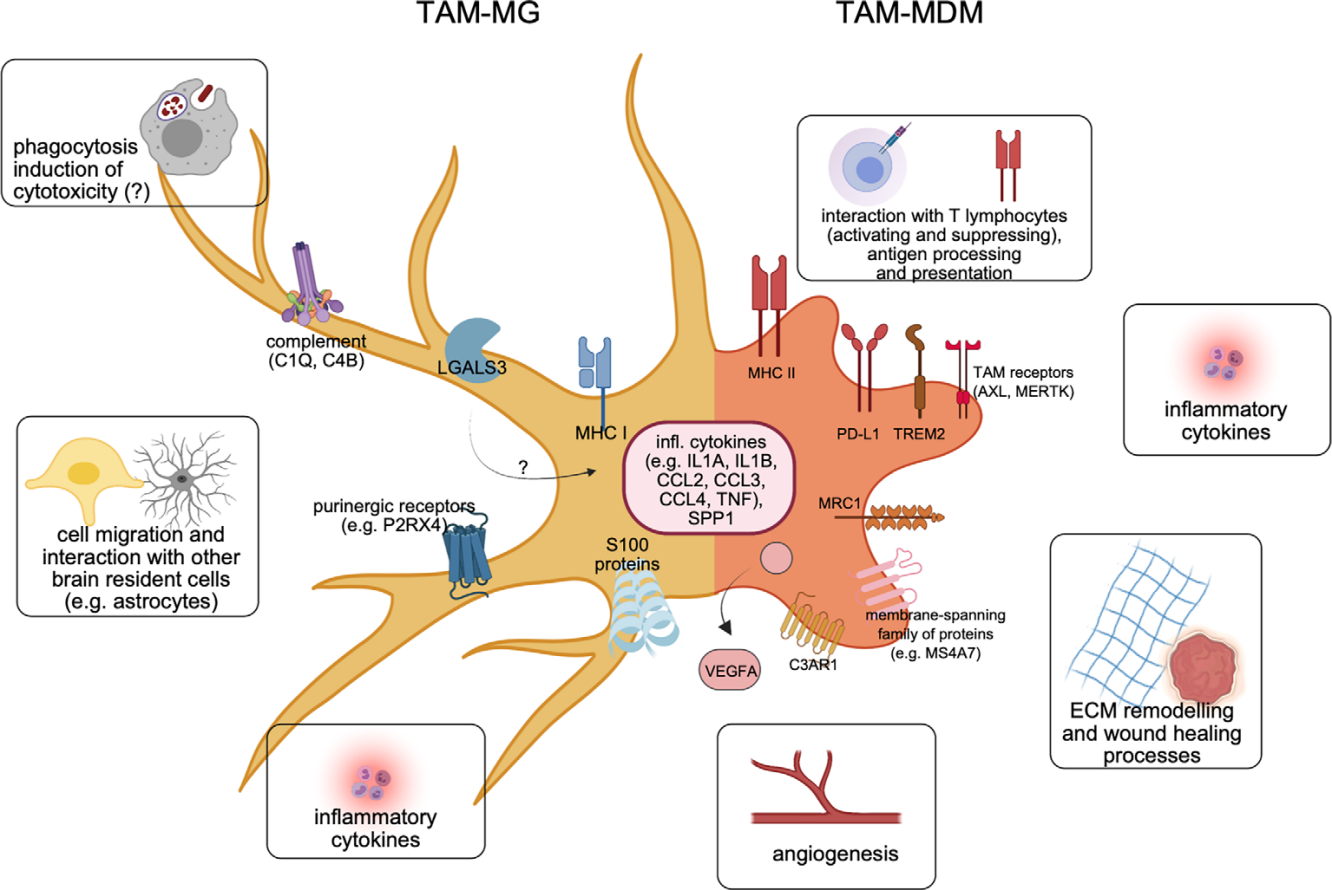
Molecular features of both major TAM populations in BrM. Recent cellular and molecular findings underline the differences of TAM-MG (left) *vs.* TAM-MDM (right) within the BrM microenvironment. Representative markers shared between TAMs from murine or human BrM are illustrated, together with major functional annotations.

Finally, in order to understand putative dichotomous functions of TAMs, and their relevance for the inflammatory TME in BrM (**Figure 3**), detailed annotation and pathway analyses of differently regulated genes were performed based on results obtained from RNA-Seq experiments (28–30, 32).

To delineate functional changes upon TAM instruction, comparative analyses of significantly differently regulated genes of TAMs *vs.* their healthy counterparts (i.e., normal MG, or blood monocytes) in breast cancer BrM (30), and lung cancer BrM (32) were performed. Together, both studies showed that altered transcriptional profiles in TAM-MG resulted in upregulation of pathways and signaling cascades associated to inflammation, regulation of cytokine production, type I interferon (IFN) signaling, cell migration and motility, and proliferation. Interestingly, TAM-MG were found to be involved in the recruitment and interaction with neutrophils, in both mouse models of BrM (30, 32), and also in human BrM (29). Concurrently, TAM-MG downregulates genes involved in housekeeping functions, such as synapse organization or regulation of neuronal organization.

TAMs derived from the periphery are more plastic than MG as described above. Thus, their altered transcriptional profiles need to be evaluated more carefully in a context-dependent manner. Compared with their normal cellular counterparts, TAM-MDMs upregulated pathways involved in inflammatory responses, cytokine production and interaction, migration, mitosis and cell cycle, and also organization of the ECM (30, 32). In addition, human BrM TAM-MDMs showed slightly higher relevance of genes related to mitosis and cell division, compared with TAM-MGs; however, staining of human BrM samples for proliferation markers indicated proliferation in both cell types (29). However, if this results from an environment promoting local proliferation, or is caused by other stimuli, e.g., prior treatment remains to be elucidated. Analyses of patient samples stratified based on treatment history will be required to gain further insight whether different intervention strategies modulate recruitment and expansion of TAM-MDMs as previously shown in a lung-to-brain model in response to radiotherapy (32). Both human TAM populations showed an enrichment for genes related to type I IFN, and NF-kB signaling (29). Not surprisingly upon entering the BrM-TME, and transitioning from monocytes, TAM-MDMs downregulated genes related to chromatin organization, and intracellular reorganization of, e.g., the cytoskeleton (30).

### Transcriptional Programs Shared in TAM-MG and TAM-MDM

In addition to lineage-restricted transcriptional programs in TAM populations, it became clear that both TAM populations also share a significant proportion of similarly regulated markers by Top marker principal component, and overlapping gene analysis in human TAMs (29), or by comparison of all differently regulated genes (DEGs) (32).

In H2030-BrM, approximately 300 DEGs were found to be commonly upregulated, and around 900 DEGs commonly downregulated between both TAM populations (32). Functional annotation of all jointly regulated markers revealed the induction of inflammatory pathways, as well as regulation of cell adhesion and cell migration. Downregulated DEGs were mostly associated to homeostatic functions in the brain, e.g., synapse organization. In contrast to the results obtained from experimental BrM models, the number of shared genes between TAM-MG and TAM-MDM was rather small in the human situation (29). The difference can potentially be explained by the fact that human RNA-seq data in this study were not stratified based on primary tumor type thereby possibly missing important similarly regulated genes. Comparison of typical inflammatory cytokines (e.g., *CCL2*, *CCL3*, *CCL4*, *IL1B*) across mice and human BrM-TAMs revealed that although most of them exhibited much higher expression levels in the BrM situation in mice, it rather were TAM-MDMs upregulating them in the human scenario (**Table 2**). RNA velocity analysis of single-cell RNA-Seq data of mouse TAMs further showed that *Il1b* and also *Tgfb* were genes similarly regulated at convergence points between TAM-MG and TAM-MDM clusters (28).

Osteopontin which is encoded by *Spp1/SPP1* is a marker usually associated to MG of early development or has been described as one key marker of all disease-associated MG (DAM) subcluster (37). In BrM, *Spp1/SPP1* was found highly expressed or upregulated in both TAM populations across species (**Table 2**), however with slightly higher expression levels in TAM-MG, similar as in primary brain tumors (34). Although a broad range of cellular functions has been assigned to osteopontin, under inflammatory conditions it most likely regulates inflammation itself *via* enhancing the recruitment of not only myeloid but also lymphoid cells (67) to the TME. Aiming to reduce or dampen inflammation within the BrM TME, osteopontin hence might represent an interesting target.

In addition to the high expression levels of the complement cascade-initiating member *C1q/C1Q* within the BrM-TME, two other inflammatory factors, *Il1a/IL1A* and *Tnf/TNF*, showed broad abundance and were highly expressed or upregulated in TAMs of murine or human BrMs (**Table 2**). Together, the presence of all three molecules strongly raises the possibility of TAM-mediated astrocyte activation towards a neurotoxic phenotype (termed A1), which partially would be sustained due to constant factor availability. This mechanism of astrocyte activation has been described in mouse models of neuroinflammatory conditions, and A1 astrocytes were found in samples of various human diseases. A characteristic marker for A1 astrocytes is the central complement component C3 (68). Interestingly, next to C1q/C1Q, other members of the complement system were apparently deregulated in BrM (32), and cross-conditional comparison revealed upregulation of, for example, *C4b/C4B* among all TAMs in mice and human (**Table 2**). While oligodendrocyte-derived *C4b* in mice has been associated to pathogenicity in an AD model (69), its functions within the TAM pool of BrM remain to be shown.

In summary, recent discoveries and previously unknown molecular insights into macrophages/microglia associated to BrM have dramatically shifted the paradigm of BrM-TAMs representing one homogeneous population. In comparison with data derived from recent “*omics*” studies involving glioma, it became clear that TAMs in brain malignancies constitute a heterogeneous mixture of resident and recruited mononuclear phagocytes, with multifaceted activation states (**Figure 3**). Moreover, each major subpopulation contributes to the inflammatory TME in a unique way, and disease-specific manner. The discovery of molecular markers present in both TAM populations or conserved between species opens novel avenues to develop targeted approaches in order to fight this deadly disease.

## Translational Aspects

TAM-targeted therapies have attracted attention as promising therapeutic strategies for a variety of different primary cancers (**Table 3**). Besides their high abundance, TAMs have been shown to critically influence tumor biology, often in a protumorigenic fashion by exerting immune-suppressive functions, and at the same time interacting with tumor cells to reciprocally support each other (35). However, in the brain, targeted approaches have to be carefully designed, in order to address modulation within specific TAM populations, without affecting resident macrophage populations (i.e., adjacent MGs) to prevent side effects.

**Table 3:** Examples of preclinical and clinical studies targeting certain TAM-related receptors/factors as mono- or combination therapies in various types of extra- and intracranial tumors.

Target	Tumor/model	Species	Treatment/drug/resource	Major effects	Study reference
C3AR	Leptomeningeal metastasis (LeptoM) models from breast and lung cancer	*M. musculus*	Nonpeptide antagonist SB290157/Santa Cruz	Prolonged survival and reduced LeptoM burden	(70)
CSF1R	Glioma	*M. musculus*	BLZ945/Novartis	Improved survival of glioma-bearing mice, tumor regression, TAM repolarization, tumor relapse observed after the period of tumor stasis	(18)
	Glioma	*M. musculus*	BLZ945/Novartis + Pi3K inhibition	Combination delays glioma relapse	(71)
			BLZ945/Novartis + IGF-1R inhibition		
	Glioma	*M. musculus*	BLZ945/Novartis + 5 × 2 Gy WBRT	Combination delays glioma relapse	(33)
	Intracerebral induced melanoma BrM	*M. musculus*	PLX3397/Selleck Chemicals	Reduction of BrM burden and BrM size	(72)
	Different primary tumors, including glioma	*H. sapiens*	Cabiralizumab (anti-CSF-1R mAB)/Five Prime Therapeutics (± nivolumab)	Ongoing study	NCT02526017
CXCR4	Adult glioblastoma (and other primary CNS tumors)	*H. sapiens*	AMD3100/Plerixafor/	Improved local tumor recurrence control	(73) (NCT01977677)
TREM2	Different primary solid tumors	*M. musculus*	Anti-TREM2 mAB clone 178	Reduced tumor growth and remodeling of myeloid landscape within the TME, enhanced immunotherapy (e.g., anti-PD-L1)	(74)
	Different primary solid tumors	*H. sapiens*	PY314/mAB against TREM2 on myeloid cells in the TME/Pionyr Immunoherapeutics	Ongoing study	NCT04691375
VEGF	Glioma	*M. musculus*	Aflibercept/VEGF-trap/Sanofi (in combination with antiangiogenic therapy, Ang-1/Ang-2 peptibody, and immunotherapy, anti-PD-1/BioXCell)	Improved survival, tumor vessel normalization, immunostimulatory reprogramming	(75)
	Breast cancer BrM	*M. musculus*	Bevacizumab/anti-VEGF mAB (in combination with anti-Ang2 L1-10)	Reduced BrM burden and permeability of blood vessels associated to BrM	(76)
	Breast cancer BrM	*H. sapiens*	Bevacizumab/anti-VEGF mAB (in combination with carboplatin)	High rate of durable objective CNS response	(77) (NCT01004172)
	Solid tumor BrM	*H. sapiens*	Bevacizumab/anti-VEGF mAB (after failure of WBRT)	About 80% of patients showed disease response, defined as stable disease or better	(78) (NCT01898130)
VISTA	Breast cancer BrM	*M. musculus*	Anti-VISTA/13F3, mAB//BioXCell (in combination with anti-PD-L1)	Reduction of BrM burden and increase of CD3+ cell abundance	(28)
	Various types of solid tumors, however exclusively without BrM	*H. sapiens*	CI-8993/anti-VISTA mAB/CURIS, Inc.	Ongoing study	NCT04475523

H. sapiens, Homo sapiens; M. musculus, Mus musculus; BrM, brain metastases; mAB, monoclonal antibody; WBRT, whole brain radiotherapy. Information on National Clinical Trial (NCT) data can be found at: www.clinicaltrials.gov.

Given the central role of CSF1-CSF1R signaling for survival and proliferation of macrophages, it is not surprising that specifically this axis has been targeted by antibodies, or small inhibitory molecules in order to reduce macrophage infiltration or deplete resident, tumor-promoting populations. Aiming to suppress tumor-promoting TAM functions, Pyonteck et al. utilized a CSF1R inhibitor in preclinical glioma models (18). Interestingly, CSF1R inhibition as monotherapy resulted in improved survival and even tumor regression, accompanied by re-education of TAMs into a rather antitumor phenotype. Long-term treatment however resulted in acquired resistance driven by IGF signaling between TAMs and tumor cells, which resulted in prolonged glioma cell survival and invasive capacities (71). Improved efficacy however can be obtained by combining CSF1R inhibition with radiotherapy in glioma (33). CSF1R inhibition was shown to reduce to breast cancer cell invasion (79) and lead to antitumor efficacy in melanoma-BrM and intracerebrally inoculated breast cancer cells (72, 80). However, therapeutic efficacy of CSF1R inhibition still needs to be carefully evaluated with regard to long-term efficacy and potential resistance mechanisms as observed in glioma.

Another strategy of limiting TAM functions within the TME includes blockade of their recruitment, *via* interfering with chemokines (81) or chemokine receptors, e.g., CCR2 or CXCR4 to inhibit general TAM recruitment (73) or by targeting newly identified markers that are implicated in the recruitment of TAM subpopulations such as CD49d (33). Given the high abundance of the anaphylatoxin receptor C3ar1/C3AR1 predominantly on TAM-MDMs of murine and human origin, blockade of this axis could also be used to inhibit monocyte/macrophage recruitment to the brain. Antibody- or small molecule-mediated inhibition of the C3-C3AR1 axis could have strong inhibitory effects and furthermore might impact the permeability of the BBB at sites of malignant inflammation. In leptomeningeal metastasis, this axis has been shown particularly important for enhancing the permeability at the choroid plexus epithelium, in order to trophically support metastasized cancer cells within the CSF (70). By interfering with the C3-C3AR1 axis, one might even trigger another antitumoral response due to blockade of the interaction of astrocytes and MG as shown by Litvinchuk et al. in a mouse model of neurodegeneration. While astrocyte-derived C3 *via* C3AR1 on MG induces proinflammatory programs *via* STAT3 signaling (82), activation of astrocytes could in part be mediated *via* C1Q plus two other cytokines, IL1A and TNF (68), which seem to be broadly present within the BrM-TME. Since astrocytes have been shown to exert multiple BrM-promoting functions (83–86) and elimination of the three aforementioned factors was beneficial in an ALS mouse model by attenuating gliosis (87), targeting one or several steps within the complement cascade seems promising and has the potential to reverse or block the immunosuppressive, cancer-permissive TME in BrM.

Although some of the markers in TAMs seem to be regulated in a more conditional manner (e.g., by different model, primary tumor entity, genetic background, species), a distinct set of genes was conservatively regulated, and similarly across TAM populations and species (e.g., MS4A proteins, TAM receptors AXL and MERTK, TREM2). While little is known about the specific functions of individual MS4A members, they might play a central role in regulating cellular functions, including cell growth, survival, and activation by serving as family of ion channels and/or adaptor proteins facilitating intracellular protein-protein interaction (41). For example, MS4A4A has been described as a key marker of BAMs (14, 15, 42) and was described as a novel M2-like marker of metastasis-associated macrophages (88). Hence future studies need to address the consequences on the inflammatory state upon interfering with, e.g., MS4A7, which was highly upregulated on both, murine and human TAM-MDMs in BrM. Given their surface exposure, MS4A members are potentially druggable by, e.g., antibodies (41).

Interestingly, within the study form Mattiola et al., the authors showed that MS4A4A interacts with a ß-glucan receptor dectin-1 in lipid rafts of the cell membrane. The dectin-1 pathway transmits intracellular signals similar to those of TREM2. Hence, TREM2 deficiency can be compensated by enhanced dectin-1 signaling (89). TREM2 signaling is essential for MG function and disease-associated phenotypes in MG can be induced by the APOE-TREM2 pathway (43). In AD, TREM2-deficient MG undergoes autophagy due to impaired mTOR signaling and metabolism (89). Since TREM2 seems to be dramatically upregulated in all TAMs across different conditions, antibody-mediated blockade of this receptor signaling pathway represents a promising approach. Within a recent study, the authors examined TREM2 function in TAMs of distinct tumor models (74). Interestingly, they found that both, mice deficient for TREM2, or antibody-mediated blockade of TREM2 signaling, resulted in delayed tumor growth. Simultaneously, the immune landscape within their model was altered including an increase of intratumoral CD8^+^ T cells, which consequently led to enhanced efficacy of anti-PD1 immunotherapy. More importantly, the authors showed ubiquitous abundance of TREM2^+^ macrophages across distinct human tumor samples, and that TREM2 expression inversely correlated with greater overall, and relapse-free survival in colorectal carcinoma and triple negative breast cancer patients (74). Given the negative correlation between MDMs and T-cell frequencies in BrM (29), it is tempting to speculate that high expression of TREM2 in TAM-MDM as seen under various BrM conditions might be an interesting candidate for combination therapies. If this furthermore leads to remodeling of the TME with enhanced T cell recruitment, also in BrM, needs to be evaluated. In summary, targeting TREM2 in combination with immunotherapy (e.g., anti-PD-L1), or radiotherapy, might represent an attractive strategy to overcome immunosuppression. It was previously shown that radiotherapy has the potential to transiently lift immunosuppressive features of the TAM pool by enhancing the recruitment of naïve monocytes/MDMs to BrM in a lung-to-brain metastasis model (32). However, acquired resistance to combined radioimmunotherapy was partially mediated by PD-L1 expression from infiltrated myeloid cells (30), which rapidly undergo tumor education. In addition to targeting the PD1-PD-L1 axis in order to enhance antitumor responses, targeting different TAM populations showed promising results in glioma and BrM models. For example, Guldner et al. inhibited the negative immune checkpoint VISTA (encoded by *Vsir*) on TAMs which similar as targeting TREM2 enhanced the CD3^+^ cell abundance within BrM leading to improved efficacy of anti-PD-L1 (28). Given the high abundance and strong expression of *Vegfa/VEGFA* in TAMs, interference with VEGF signaling could furthermore lead to enhanced antitumor responses, as shown in a triple treatment approach of murine glioma (75). The authors blocked the angiogenic factors VEGF and ANG-2 in combination with PD-1, which resulted in extended survival of mice compared with anti-VEGF as monotherapy (75). However, targeting VEGF in BrM is not indicated for every primary tumor type which gives rise to BrM. While double inhibition of VEGF and ANG-2 reduced BrM burden in preclinical models of breast-to-brain metastasis (76), VEGFA inhibition can induce long-term dormancy in lung-to-brain metastasis (90). For breast-to-brain metastasis patients, combination of VEGFA inhibition with bevacizumab in combination with carboplatin resulted in a high rate of durable responses (77). Moreover, VEGFA inhibition resulted in a 25% disease response rate in 80% of solid cancer patients with current brain metastasis that failed whole-brain radiotherapy (78).

In summary, novel targeted therapeutic approaches need to be carefully evaluated in a context-specific manner upon spatiotemporal determination of leukocytic subsets within the TME. This is particularly important for targeting specific phenotypic features of TAMs, but at the same time spare homeostatic features of adjacent, non-BrM-associated populations. Furthermore, it will be critical to evaluate to which extent altered gene expression also translates into altered protein abundance, which in addition requires evaluation on a spatial level. In combination with standard therapy, targeting distinct TAM subsets represents a promising strategy. Combination therapies are expected to induce synergy by on the one hand repressing tumor-promoting traits, and on the other hand lifting immunosuppression, thereby enhancing antitumor immunity.

## Concluding Remarks

Driven by technical advances, as well as scientific and clinical interest in understanding cellular and molecular landscapes in health and disease, recent research has resulted in tremendous insight into the heterogeneity of TAM of primary and secondary BrMs.

RNA sequencing, multiplexed flow, and mass cytometry revealed the dichotomous nature of TAMs in BrM, wherein resident microglia as well as recruited monocyte-derived macrophages represent the two major populations and contribute significantly to the entire immune cell landscape. Although both TAMs quantitatively differentially contribute to the local TAM pool and populate different niches within the TME, their phenotypic changes occur early upon disease-specific instruction in a highly plastic manner. Together, both TAM populations contribute to the establishment of an immunosuppressive and tumor-promoting environment in BrM. In order to evaluate the applicability of novel targeted approaches, further research needs to determine molecular pattern in spatiotemporal resolution. Detailed mechanistic understanding how standard therapy can be used as an immune modulator in addition to the identification of transcriptional programs that drive disease-promoting states in TAMs to provide scientific rationale for the development of improved therapeutic avenues against BrM is needed.

## Author Contributions

MS and LS conceptualized and wrote the manuscript. All authors contributed to the article and approved the submitted version.

## Funding

Research in the lab of LS is supported by institutional funds from the Georg-Speyer-Haus jointly funded by the German Federal Ministry of Health and the Ministry of Higher Education, Research and the Arts of the State of Hesse (HMWK), as well as grants from the German Cancer Aid (Max-Eder Junior Group Leader Program 70111752) and German Research Foundation (SE2234/3-1).

## Conflict of Interest

The authors declare that the research was conducted in the absence of any commercial or financial relationship that could be construed as a potential conflict of interest.

## Publisher’s Note

All claims expressed in this article are solely those of the authors and do not necessarily represent those of their affiliated organizations, or those of the publisher, the editors and the reviewers. Any product that may be evaluated in this article, or claim that may be made by its manufacturer, is not guaranteed or endorsed by the publisher.
